# Solid Peroxy Compounds as Additives to Organic Waste for Reclamation of Post-Industrial Contaminated Soils

**DOI:** 10.3390/ma14226979

**Published:** 2021-11-18

**Authors:** Angelika Więckol-Ryk, Maciej Thomas, Barbara Białecka

**Affiliations:** 1 Department of Risk Assessment and Industrial Safety, Central Mining Institute, Plac Gwarków 1, 40-166 Katowice, Poland; 2 Faculty of Environmental and Power Engineering, Cracow University of Technology, Warszawska 24, 31-155 Cracow, Poland; 3 Department of Environmental Monitoring, Central Mining Institute, Plac Gwarków 1, 40-166 Katowice, Poland; bbialecka@gig.eu

**Keywords:** calcium peroxide, sodium percarbonate, calcium hydroxide, poultry manure, land reclamation, phytotoxicity test

## Abstract

Solid peroxy compounds have been increasingly applied for the removal of organic pollution from contaminated groundwater and soil due to their ability to release oxygen and hydrogen peroxide. The influence of two solid peroxy compounds (sodium percarbonate, 2Na_2_CO_3_·3H_2_O_2_ and calcium peroxide, CaO_2_) with poultry manure (PM) added to contaminated soil on the growth of the tested plants (*Sinapis alba, Lepidium sativum L.* and *Sorghum bicolor L. Moench*) and the quality of soil water leachates was investigated. A series of experiments involving the addition of CaO_2_ and 2Na_2_CO_3_·3H_2_O_2_ at the dose of 0.075 g/g PM improved the growth of tested plants. The conducted study indicated that the use of peroxy compounds not only removed pathogens from livestock waste, but also improved the quality of plant growth. The calculated factors for the growth of roots (GFR) and growth of shoots (GFS) in soils treated with a mixture of peroxy compounds and PM were higher than in soils treated only with PM. The physicochemical analysis of soil water leachates indicated that solid peroxy compounds may be a promising alternative compared to the currently used hygienizing agent such as calcium hydroxide (Ca(OH)_2_). Solid peroxy compounds increased the bioavailability of components necessary for proper seed germination and plant growth (N, P, K, Ca, Mg and S). In most of the studied cases, the obtained plant shoot and root growth rates were higher for soil mixtures containing organic waste deactivated by biocidal compounds, compared to soils that contained only poultry manure.

## 1. Introduction

Solid peroxy compounds such as calcium peroxide (CaO_2_) and sodium percarbonate (2Na_2_CO_3_·3H_2_O_2_) are widely studied green oxidants that have potential for use in various research fields. In recent years, alkaline peroxides and percarbonates have been applied to remove organic and inorganic pollutants especially from groundwater and sewage sludge [1,2,3,4,5]. Many researchers have confirmed that the addition of the peroxy reagents mentioned significantly affects the process of soil bioremediation [6,7].

However, the disposal of hazardous compounds by solid peroxides and percarbonates is not their only successful application. Sodium percarbonate is used in the cleaning industry as a bleaching agent [8] and is much safer than the concentrated hydrogen peroxide solution (H_2_O_2_). Additionally, studies carried out by many authors [1,9,10] showed the potential use of sodium percarbonate and calcium peroxide as effective alternatives to liquid H_2_O_2_ for the treatment of chlorinated aromatic hydrocarbon-contaminants in soils such as 4-chloronaphtol, 2,4-dichlorophenoxyacetic acid, trichloroethene or polychlorinated biphenyls compared to Fenton’s reagent.

In addition, the alkaline peroxy compounds show a microbiocidal effect on many harmful microorganisms present not only in groundwater but also in soils and even organic fertilizers. In our previous studies [11,12], the process of chicken manure microbial deactivation with calcium peroxide and sodium percarbonate as the biocidal agents was successfully optimized. The obtained results proved that the addition of 8 wt.% of CaO_2_ or 7.5 wt.% of 2Na_2_CO_3_·3H_2_O_2_ into the fresh chicken manure may decrease the number of harmful bacteria *Eschericha coli* (*Enterobacteriaceae* family) from 10^8^ cfu/g to an acceptable level below 10^3^ cfu/g, with the latter being in accordance with Polish law [13]. The interesting antimicrobial activity of CaO_2_ was studied previously by other researchers [14,15,16].

Sodium percarbonate and calcium peroxide are the most extensively studied chemical compounds, which show disinfecting properties through the generation of hydrogen peroxide. In a moist environment, 2Na_2_CO_3_·3H_2_O_2_ and CaO_2_ decompose to H_2_O_2_ releasing active oxygen, according to the following formulas (1–3):

2Na_2_CO_3_ · 3H_2_O_2_ → 2Na_2_CO_3_ + 3H_2_O_2_ (1)

CaO_2_ + 2H_2_O → Ca(OH)_2_ ↓ + H_2_O_2_ (2)

2H_2_O_2_ → 2H_2_O + O_2_ ↑ (3)

The content of active oxygen in commercial solid peroxides is in the range of 17–18 wt.% for CaO_2_ and about 13 wt.% for 2Na_2_CO_3_·3H_2_O_2_ [17]. Calcium peroxide is characterized by a slow decomposition rate, which allows the generation of oxygen for prolonged durations. Due to its slow solubility in water (1.65 g/L), the rate of oxygen release on the soil condition from CaO_2_ is estimated to be in the of range 3–9 months [18] in comparison to sodium percarbonate, which is highly soluble in water 150 g/L [19]. Another advantage of calcium peroxide is the fact that it is easy to produce in the field by heating lime with hydrogen peroxide.

Sodium percarbonate is described by the US Environmental Protection Agency as an algicidal and fungicidal compound for use in commercial greenhouses, gardens or landscapes [20]. It has advantages comparable to liquid H_2_O_2_ and was approved for the control of blue-green algae in lakes, ponds and drinking water reservoirs [19].

Solid peroxy reagents can also be applied in agriculture to stimulate seed growth and their earlier germination. For instance, calcium peroxide is used in coatings to supply oxygen during germination to rice seeds. Large-scale commercial utilization of seed coating for field-scale precision agriculture began in the 1960s and is constantly increasing [21]. Seed coating provides an inexpensive and efficient technology improving germination and the quality of crops. Some studies found that seed pelleting with CaO_2_ promoted the germination of rice directly sown into flooded soil and improved plant development [22,23]. The effect of calcium peroxide coating of rice seeds on seedling establishment and growth in an oxygen-depleted environment was also studied by Biswas et al. [24]. However, it was concluded that the efficacy of this process depended, among others, on the soil type and its source.

For the reclamation of post-industrial soils and those poor in macronutrients, there arises the need to use fertilizers that have a decisive influence on plant growth.

Poultry manure (PM) from the production of layers, broilers or turkey is one of the organic fertilizers that reveal a high concentration of valuable nutrients such as nitrogen (N), potassium (K), phosphorus (P), sulfur (S), calcium (Ca) or magnesium (Mg) [25,26]. However, there is a growing need for improved PM content for minimizing the environmental risk following its application. Poultry manure may contain excessive amounts of heavy metals including arsenic (As), cobalt (Co), copper (Cu), iron (Fe), manganese (Mn), selenium (Se), nickel (Ni), lead (Pb) or zinc (Zn) [27], which are supplied to the soil in the fertilization process and may significantly increase their concentration in the soil and groundwater. Furthermore, PM is the most serious source of hazardous pathogens, such as bacteria, viruses, fungi and yeast, with the most common ones including the bacteria *Salmonella, Campylobacter, Yersinia*, *Listeria monocytogenes* and *E. coli* [28,29,30,31,32]. The microorganisms present in organic waste may be transported from the soil surface to the groundwater and then to plant roots. The negative impact of animal manure on the environment and human health has been reported by Unc and Gross [33]. For that reason, before PM is applied as a fertilizer to the soil, its biological, chemical or physical deactivation is required [34,35]. The most popular technique of PM deactivation is composing it with one of the calcium compounds (CaO, Ca(OH)_2_ or CaCO_3_) [36]. However, during this process, pH reaches up to 12, which leads to the rapid release of nitrogen by converting it to gaseous ammonia. Furthermore, under the influence of lime, phosphorus stabilizes in the soil creating forms unavailable to plants [15]. To avoid these losses, a novel approach of use other than traditional lime compounds such as calcium peroxide and sodium percarbonate has been proposed. Both are considered green oxidizers, which are rapidly degraded in soil, and are safe for the natural environment and do not bioaccumulate.

The aim of this investigation was to determine the influence of poultry manure treated with solid peroxy compounds added to contaminated soil on the growth and quality of the tested plants and the quality of soil water leachates.

## 2. Materials and Methods

### 2.1. Poultry Manure and Soils

Poultry manure was collected from commercial poultry houses of laying hens, located in the Silesia region in Poland (N 50°6′26.154″, E 18° 43′53.977″). The raw reference sample of poultry manure used for the experiments was denoted as PM. Before being mixed with soil samples, the fresh poultry manure had been air dried in a laboratory for 7 days (21 ± 1 °C) and crushed to pass through a 2 mm sieve.

Two contaminated soil samples from different industrial complex surrounding areas i.e., the Zinc Smelting Plant in Miasteczko Śląskie (S1) and the Non-Ferrous Metal Plant in Szopienice (S2), were used in this investigation. Soil samples were collected to a depth of 0–15 cm, air-dried at stable room temperature (21 ± 1 °C), crushed to pass through a 2 mm sieve and then stored in plastic containers in ambient temperature. The physicochemical analysis of the poultry manure and soils was described in our previous papers [37] and presented in Table 1.

Additionally, OECD artificial soil, defined as a mixture of 75% fine quartz sand (50% particles 0.05–0.2 mm), 20% kaolin clay (kaolinite content preferably above 30%) and 5% finely ground Sphagnum peat was used as the control soil (C) in the tests, according to the international standard EN 18763 [38].

### 2.2. Chemical Reagents

Two solid peroxy compounds i.e., technical grade (available oxygen, ≥12.8%) sodium percarbonate (2Na_2_CO_3_·3H_2_O_2_, Brenntag, Poland) and technical grade (78.1%) calcium peroxide (CaO_2_), (Ixper^®^ 75C, Solvay Chemicals International S.A., Brussels, Belgium) were applied as the green deactivation agents. Additionally, calcium hydroxide (Ca(OH)_2_, Chempur, Piekary Śląskie, Poland) was used as a traditional hygienizing agent. The chemical reagent was denoted as B1 (sodium percarbonate), B2 (calcium peroxide) and B3 (calcium hydroxide).

### 2.3. Preparation of Soil Blends

A weight of 20.000 ± 0.001 g of fresh poultry manure was mixed with 1.500 ± 0.001 g of inorganic peroxy compounds i.e., calcium peroxide (B1) or sodium percarbonate (B2) as well as with 1.000 ± 0.001 g of calcium hydroxide (B3) as the conventional hygienizing agent. The mixtures were then air dried at room temperature for 7 days (24 ± 1 °C), crushed and passed through a 2 mm sieve and then mixed with 1.000 ± 0.001 kg of soils S1 or S2 to obtain the soil blends. The soil mixture treated only with poultry manure was denoted as PM. The addition of biocidal agents B1-B3 to soil with poultry manure PM was expressed as follows: PM (B1), PM (B2) and PM (B3).

A dose of PM (2 wt.%) was calculated for the treatment of 10 t organic fertilizer in one hectare of soil.

The amount of chemical reagents B1, B2 and B3 corresponded to the doses necessary to reduce pathogens in fresh PM below the level of 1000 cfu/g [13]. Doses for B1 (7.5 wt%) and B2 (8.0 wt.%) were calculated according to our previous studies [11,12] whereas the amount of B3 (5.0 wt.%) corresponded with the dose used in the conventional hygienizing process [39].

### 2.4. Analytical Procedures

Soil samples and their blends were placed into 500 cm^3^ bottles and added to deionized water in a soil-to-water ratio of 1:10 (on dry weight) according to standard EN 12457-4 [40]. After being shaken on a rotary mixer (ROTAX 6.8, Velp Scientifica Srl, Usmate, Italy) at a speed of 40 rpm for a period of 8 h, the samples were centrifuged at 15,000 rpm for 10 min (Centrifuge 5810, Eppendorf, Hamburg, Germany) and filtered using Whatman 0.45 µm filters (GE Healthcare, Chicago, IL, USA).

The content of macronutrients (Ca, Mg, K, P, S) and selected metals (As, Cd, Cr, C, Ni, Pb, Se, Zn) in water extracts was determined by the ICP-OES method (Perkin Elmer Optima 5300DV ICP-OES analyzer, Perkin Elmer, Encino, LA, USA). The content of total organic carbon (TOC) was determined by an elemental analyzer with infrared detection (TOC-L CPH, Shimadzu, Kyoto, Japan) whereas the content of total nitrogen was determined with the high-temperature infrared chemiluminescence detection (TNM-L Shimadzu, Kyoto, Japan). The pH-value and electrical conductivity (EC) of water extracts were measured by the pH-meter (CPC-411, Elmetron, Zabrze, Poland) with the combination electrode (IJ44AT, Elmetron, Zabrze, Poland), respectively.

The availability of phosphorous in soil samples and their blends was determined with the Egner–Riehm method [41]. A total weight of 2.000 ± 0.001 g of soil samples on dry weight were added to 100 mL of a 0.04 mol/L calcium lactate solution with hydrochloric acid after buffering to pH 3.55 ± 0.05. The samples were stirred for 1.5 h at a speed of 40 rpm in a rotary mixer (Trayster Digital, IKA Werke GmbH & Co. KG, Staufen, Germany), then centrifuged (Centrifuge 5810, Eppendorf, Hamburg, Germany) and the obtained extracts were filtrated under pressure using Whatman 0.45 µm filters (GE Healthcare, Chicago, IL, USA).

The availability of phosphorous in soil samples and their blends was determined with the ammonium molybdate spectrophotometric method, based on standard EN ISO 6878 [42], after pressure mineralization (K_2_S_2_O_8_, 120 °C, 30 min) with Nanocolor^®^ tube test P15 tests (0.3–15.0 mg/L) and a spectrophotometer (Nanocolor 500D, Macherey-Nagel GmbH & Co. KG, Düren, Germany).

### 2.5. Phytotoxicity Tests

The tests were performed according to the modified procedure based on the European standard EN ISO 18763 [38], which measures the decrease or increase in the young roots and shoots after a few days of exposure of seeds in comparison to the control soil. To determine phytotoxicity of the soil blends with biocide agents, three test organisms were chosen i.e., white mustard (*Sinapis alba L.*), garden cress (*Lepidium sativum L.*) and sorgo (*Sorghum bicolor L. Moench*). A 35.000 ± 0.001 g soil sample, or its blend, was placed in sterile plastic Petri dish (9 cm diameter) and mixed with 20.0 ± 0.1 mL of distilled water. Each dish was then lined with filter paper of 87 g/m^2^ (grade 292, Munktell, Ahlstrom Munksjö, Finland) and left until saturated with moisture. Then, 10 seeds of the tested plant were placed out on filter paper and closed with the lid. To determine the effect on germination, the seeds were incubated in darkness at 25 ± 1 °C for 72 h. The length of roots and shoots were measured as the main parameters of the growth of the plants with an accuracy of ± 0.1 cm.

The value of the stimulation of plant growth was calculated as the growth factor of the root (GFR) and the growth factor of the shoot (GFS) according to Equations (4) and (5):

(4) $$GFR=\frac{R_{S}-R_{C}}{R_{S}}\cdot 100\%\;$$

where *R_S_* is the average length of roots on the tested soil and *R_C_* is the average length of roots on the control soil, and

(5) $$GFS=\frac{S_{S}-S_{C}}{S_{S}}\cdot 100\%\;$$

where *S_S_* is the average length of shoots on the tested soil and *S_C_* is the average length of shoots on the control soil.

All experiments were conducted in triplicate.

### 2.6. Statistical Analysis

Statistical analysis of the experimental data was performed using Statistica 13 (StatsSoft, Poland). The data represented mean ± standard deviation. The differences between many groups were compared using an analysis of variance one-way ANOVA and the post-hoc Tukey’s HSD (Honestly Significant Difference) test. A probability level of *p*-value less than 0.05 was considered statistically significant.

The determination of metals and non-metals in the soil water leachates using the ICP-OES method was performed with the level of uncertainty of 15%, a coverage factor of 2 and a significance level of 95%.

## 3. Results and Discussion

### 3.1. Physicochemical Characteristic of Soil Leachates

**Figure 1:**
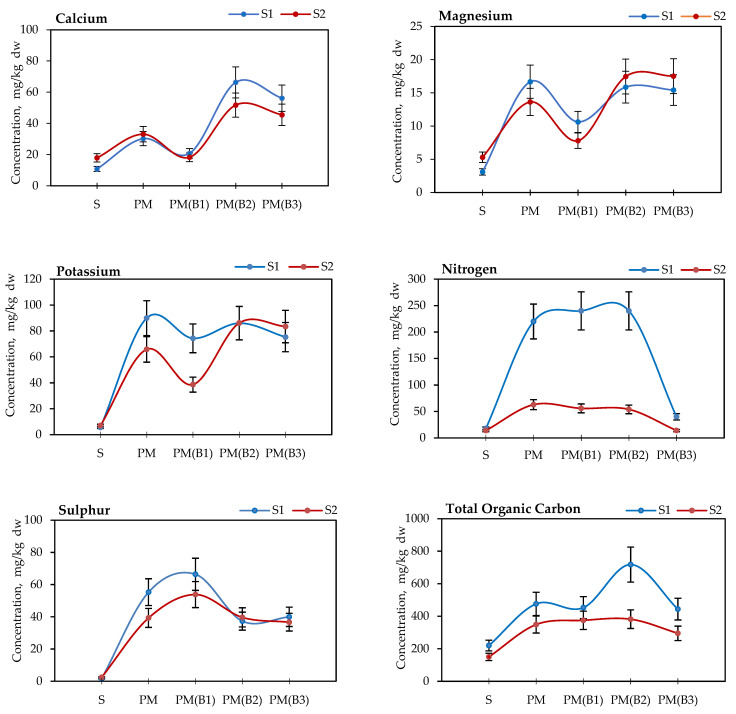
Concentration of macronutrients in soils and their blends treated with 2 wt.% poultry manure (PM) and biocidal agents: B1—calcium peroxide, B2—sodium percarbonate, B3—calcium hydroxide, S—soils without treatment (S1 or S2).

The results of physicochemical parameters of the soils and their blends with the addition of poultry manure and biocidal agents (B1, B2 and B3) in water extracts are presented in Figure 1 and Table 2.

The analysis of the chemical composition of the water extracts of the soil mixtures showed reproducible trends of increases and decreases in the concentrations of individual elements. As shown in Figure 1, the addition of 2 wt.% PM to the tested soil samples increased the content of macronutrients (Ca, N, K, P, Mg, S) in a form bioavailable to plants. Moreover, the analysis showed that the addition of B1 stimulated the bioavailability of phosphorus and sulphur, while the addition of B2 significantly increased the amount of calcium, potassium, magnesium and nitrogen in the water extracts.

In the case of the B1 and B2 additives, opposite trends of increasing elemental concentrations were observed, which may affect the method of selecting the appropriate biocide agent for different types of soils as well as the type of organic waste applied. The application of calcium hydroxide (B3) as a hygienizing agent indicated a decrease in the availability of nitrogen and sulphur in the water extracts of soils. The highest losses were observed for nitrogen, whose concentrations in PM (B3) samples amounted to 40 mg/kg dw (S1) and 14 mg/kg dw (S2), respectively, and were comparable to the concentrations in test soils of 18 mg/kg dw (S1) and 14 mg/kg dw (S2). The results obtained confirm those of the studies conducted by other researchers. The concentration of nitrogen in the leachates obtained from the soil with organic fertilizer was also observed by other researchers. Ghaly and Singh [43] showed that after long-term soil application of cattle manure, nitrogen was lost through ammonia volatilization, from 33 to 85 kg/ha. Moreover, the loss of ammonia in poultry manure may result in a decrease in the pH value [44]. It also proved that the application of soil lime for the hygienization of organic waste led to the strong alkalinization process that occurs with ammonia emissions and a decrease in the nitrogen content [39].

**Figure 2:**
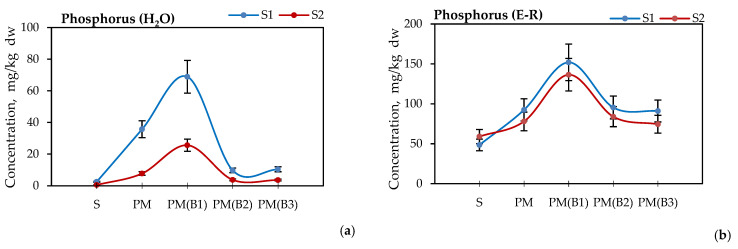
Concentration of phosphorus in soils and their blends treated with 2 wt.% poultry manure (PM) and biocidal agents B1—calcium peroxide, B2—sodium percarbonate, B3—calcium hydroxide, S—soils without treatment (S1 or S2); (**a**) extraction with water, (**b**) extraction with calcium lactate (Egner–Riehm method).

Figure 2a,b shows the results of phosphorus content in the plant-available form for the water-soluble fraction and in the buffered calcium lactate solution, according to the Egner–Riehm method (E-R) [41].

The analysis of phosphorus content by water extraction and the Egner–Riehm method showed a visible effect of sodium percarbonate addition on the increase in the bioavailable phosphorus concentration. Water extraction yielded 68.91 mg/kg dw for soil S1 and 35.73 mg/kg dw for soil S2. These values were, on average, seven times higher than deactivated samples B2 i.e., 9.72 mg/kg dw for S1 and 3.74 mg/kg dw for S2 and hygienized samples B3 i.e., 10.41 mg/kg dw for S1 and 3 mg/kg dw for S2. When extracted with a calcium lactate solution, the phosphorus concentration in PM (B1) samples was less than twice that of PM (B2) samples i.e., 95.5 mg/kg dw for S1 and 84.0 mg/kg dw for S2 and of PM(B3) samples i.e., 91 mg/kg dw and 74.5 mg/kg dw. The increase in phosphorus concentration in B1 samples may be related to the decomposition of organic phosphates contained in soil and PM i.e., humus compounds, phosphoproteins, phospholipids, phosphoglycosides, phosphoamines and nucleic acids [45], due to a rapid reaction of their oxidation. The Egner–Riehm method uses an extraction solution whose pH is acidic. Under such environmental conditions, plants best utilize phosphorus bound to iron and aluminum forms [46].

The presence of increased amounts of calcium in samples PM (B2) and PM (B3) inhibited the decomposition of humus and organic compounds. The apparent decrease in the phosphorus concentration in PM (B2) samples compared to PM (B1) samples may also be related to the formation of an insoluble phosphorus compound i.e., hydroxyapatite Ca_5_(PO_4_)_3_OH, which is formed by the reaction of Ca(OH)_2_ with phosphate ions, and may occur in two steps, as shown in Equations (6) and (7) [47]:

CaO_2_+2H_2_O→Ca(OH)_2_+H_2_O_2_ (6)

5 Ca(OH)_2_ + 3 H_3_PO_4_→Ca_5_(PO_4_)_3_OH↓+9 H_2_O (7)

Hydroxyapatite is a well-known phosphorus-containing mineral used in the immobilization of heavy metals in soil contamination with heavy metals i.e., Cd, Cu, Ni, Pb and Zn [48,49]. The immobilization mechanism involves the exchange of heavy metal ions with Ca^2+^ ions on the surface of phosphate minerals, which leads to the formation of amorphous solids. The use of phosphorus-containing additives, such as poultry manure, is particularly recommended when Pb immobilization is required [50,51]. The application of hydroxyapatite in the form of phosphorus amendments promotes the formation of pyromorphite Pb_5_(PO_4_)_3_X where X is F, Cl, Br and OH stable Pb phosphate minerals [52].

However, caution is recommended in the case of As, whose bioavailability increases in the presence of phosphates [53].

The results of the study of the heavy metal content presented in Table 2 clearly indicate that, compared to the samples containing sodium percarbonate B1, the addition of calcium compounds B2 and B3 visibly reduced the content of Cu, Cd, Pb and Zn in the aqueous leachate samples.

The contents of As, Pb and Cu in PM (B1) samples were two and three times higher than in PM (B2) samples. The zinc content of PM (B1) samples was slightly higher than the others, while the chromium, cadmium and nickel contents of PM (B1) samples were significantly higher for soil S1.

In addition, soil samples deactivated with sodium percarbonate PM (B1) presented a higher electrical conductivity value, which may have influenced the inhibition of the growth of test plants, especially *S. alba*, which is an organism very sensitive to the degree of salinity. The analysis showed that the pH of the soil samples tested varied from neutral to slightly alkaline. Soil pH is one of the factors determining the mobility of heavy metals in soils and affects the balance of sorption and desorption processes of metal cations. According to Karczewska [54], in general, the solubility of heavy metals in soil is low in the range of neutral and alkaline pH and increases with a decreasing pH value. Cd compounds are considered to be the most soluble (pH 6.5), followed by Zn, Cu and Ni. Cr, Pb and Hg compounds are characterized by the weakest mobility in soil. In our study, the highest pH value was obtained for PM (B1) samples at 7.28 for S1 and 7.68 for S2, which may have caused an increase in the solubility of metals in aqueous leachates. Nevertheless, the reason for the increased content of soluble forms of metals in PM (B1) samples was their oxidation under the influence of rapid H_2_O_2_ release. This reaction is commonly used in remediation processes of soils contaminated with organic compounds.

The highest increase in TOC concentration was observed in PM (B2) samples 718 and 382 mg/kg dw, where H_2_O_2_ was slowly released. An example of the reduction of the organic carbon concentration presented by Walawska et al. [17] showed that a decrease in the soil organic carbon concentration after the addition of 1–5 g CaO_2_/kg was observed after only a few days, and the efficiency of the process of oxidation of organic compounds was estimated at 240 mg C per kg of soil.

Based on De La Calle et al. [55], the optimum remediation conditions using sodium percarbonate depend on many parameters, such as soil type, temperature and peroxide concentration. Therefore, the authors suggested that specified studies should be conducted for each real soil used.

### 3.2. Statistical Analysis

The results of the analysis of variance of one-way ANOVA test on the length of roots and shoots of the tested plants is presented in Table 3 and Table 4.

The analysis indicated that both application of PM and the biocidal agents had a substantial effect on plant growth. The growth of the tested plants increased significantly (*p* < 005) with the use of soil and the additive PM and biocide agents B1-B3. The results for *Sorghum b.* showed that the length of the shoot was significantly less (*p* = 0.04 for S1 and *p* = 0.03 for S2) in comparison with *S. alba* and *L. sativum.*

**Figure 3:**
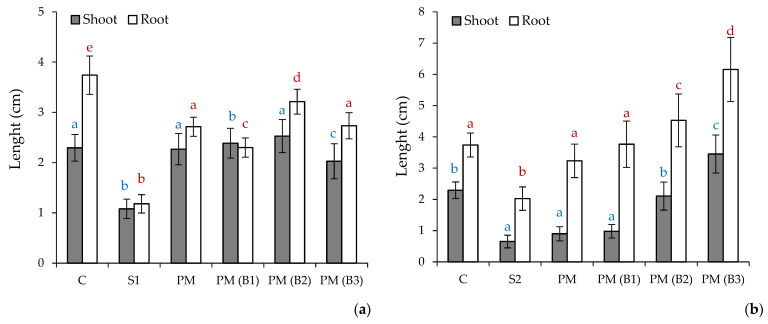
Data represent the average value ± the standard deviation (*n* = 15) of the length of roots and shoots of the *Sinapis alba L.* seeds tested on soil S1 (**a**) and S2 (**b**). Different letters indicate no significant differences Tukey’s test HSD (*p* > 0.05); C—reference soil.

The results of Tukey’s multiple range test are presented in Figure 1, Figure 2 and Figure 3. The obtained results in the compared groups were not significantly different from each other (*p* = 0.05)

### 3.3. Results of Phytotoxicity Tests

**Figure 4:**
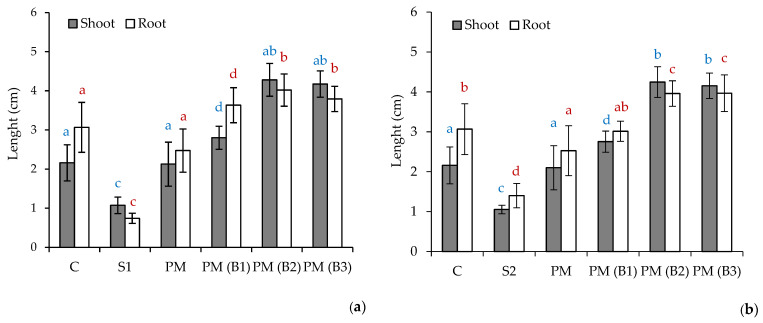
Data represent the average value ± the standard deviation (*n* = 15) of the length of roots and shoots of the *Lepidium sativum L.* seeds tested on soil S1 (**a**) and S2 (**b**). Different letters indicate no significant differences Tukey’s test HSD (*p* > 0.05); C—reference soil.

**Figure 5:**
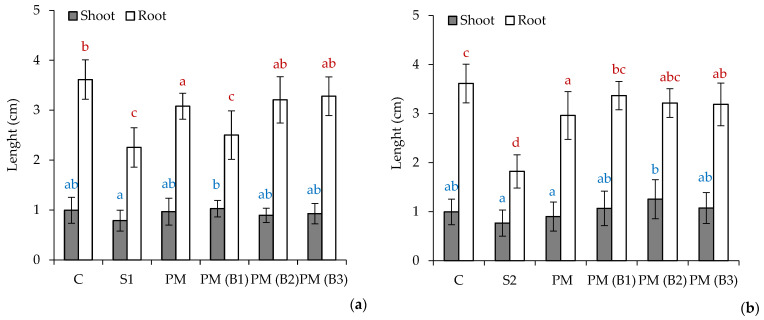
Data represent the average value ± the standard deviation (*n* = 15) of the length of roots and shoots of the *Sorghum bicolor L. Moench* seeds tested on soil S1 (**a**) and S2 (**b**). Different letters indicate no significant differences Tukey’s test HSD (*p* > 0.05); C—reference soil.

The results of the measurements of the root and shoot length for the examined sprouts of the plants *Sinapis alba L. (S. alba), Lepidium sativum L. (L. sativum)* and *Sorghum bicolor L. Moench (S.bicolor.)* are illustrated in Figure 3, Figure 4 and Figure 5.

**Figure 6:**
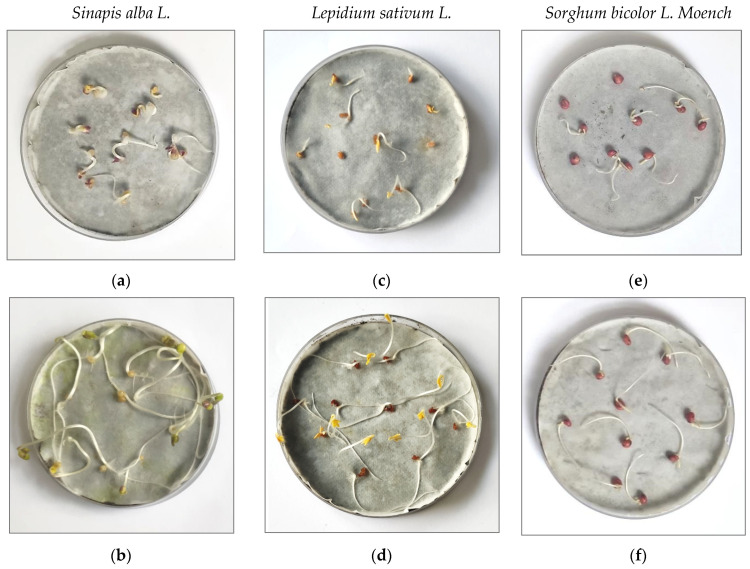
Exemplary photos of plants after germination test: (**a**) *Sinapis alba L.* on soil S2; (**b**) *Sinapis alba L.* on soil S2 treated with PM(B2) on soil S2; (**c**) *Lepidium sativum L.* on soil S1; (**d**) *Lepidium sativum L. on soil S1* treated with PM(B2); (**e**) *Sorghum bicolor* on soil S2; (**f**) *Sorghum bicolor* on soil S2 treated with PM(B1).

Photos of the Petri dishes after germination of the tested plants on the tested soil and their blends are presented in Figure 6.

The obtained experimental data indicate varied levels of phytotoxicity of the tested soils and their mixtures in comparison to the control soil (C).

The average root length of the plants *L. sativum.*, *S. alba* and *S. bicolor* on the C soil amounted to 3.07, 3.74 and 3.61 cm, respectively, whereas the average length of the shoot of the said plants reached the values of 2.16, 2.29 and 0.99 cm, respectively.

In soil S1 from the Zinc Smelter in Miasteczko Śląskie, plant growth was inhibited in comparison with soil C, with mean root and shoot lengths of 0.74 and 1.07 cm for *L. sativum*, 1.18 and 1.08 cm for *S. alba* and 2.25 and 0.79 cm for *S.* bicolor, respectively.

The S2 soil from Nonferrous Metals Steelworks in Szopienice showed inhibition of plant growth compared to reference soil C, with mean root and shoot lengths of 1.40 and 1.05 cm for *L. sativum,* 2.03 and 0.62 cm for *S. alba* and 1.82 and 0.77 cm for *S.* bicolor, respectively.

These results indicate the high toxicity of S1 and S2 test soils due to nutrient deficiency and the relatively high heavy metal content. As reported in our previous study [37], the series of metal concentrations were Zn > Pb > Mn > Cu > Cd > Al > Cr > Fe > Ni > Co for soil S1 and Zn > Pb > Mn > Cu > Cr > Cd > Al > Fe > Ni > Co for S2. The zinc concentration for S1 was 6920.1 mg/kg dw, which indicates that it exceeded its permissible value specified in the Regulation of the Minister of Environment by six times in the manner of conducting the assessment of land surface pollution [56] for soil group III, i.e., 1000 mg/kg dw. In addition, soil S1 also exceeded the permissible concentration of lead 942.5 mg/kg dw and cadmium 35.2 mg/kg dw, with limit values of 500 and 10 mg/kg dw. Soil S2 showed a slight exceedance of the permissible concentration of cadmium i.e., 12.1 mg/kg dw. Soil S2 had lower major nutrient contents compared to soil S1 for Ca (0.6 and 1.6 g/kg dm), K (4.8 and 7.2 g/kg dm), Mg (0.2 and 0.7 g/kg dm) and P (0.2 and 0.3 g/kg dm), respectively. The addition of PM at 2% by weight to the soil substrates tested resulted in growth stimulation of all test plants. A twofold increase in root and shoot growth was recorded for *L. sativum* and *S. alba.*

The study of the effect of the addition of biocidal and hygienizing compounds showed a positive effect of their influence on the germination process and the growth of the test plants. The most effective additive was calcium peroxide (B2), which increased shoot and root growth of *L. sativum* fourfold, *S. alba* more than twofold and the root growth of *S. bicolor* by one. The lowest growth of *S. bicolor* may have resulted from the fact that this plant does not require fertile soils, and the measured length of its root on soils S1 and S2 with PM(B2) was 3.21 cm, similar to the control soil where it reached 3.61 cm. Sorghum is mainly grown on low-potential, shallow soils with a high clay content, which are usually not suitable for the production of other grain crops. *S. bicolor* is more tolerant of alkaline salts and can therefore be successfully cultivated on soils with a pH (measured in KCl) between 5.5 and 8.5 [57]. Phytotoxicity tests indicated that the addition of sodium percarbonate slightly stimulated the root and shoot growth of *L. sativum* and *S. bicolor.* In the case of *S. alba,* a slight inhibition of root growth was obtained in soil PM(B1) compared to PM i.e., 2.30 cm and 2.71 cm, respectively. In the S2 soil, the root growth of this plant was slightly higher for PM(B1) than for PM i.e., 3.77 cm and 3.23 cm, respectively. This may be related to the lower content of metals in S2 soil, which under the influence of H_2_O_2_ released from sodium percarbonate are converted into bioavailable forms for plants. The best results for *S. alba* on the S2 soil were obtained for the sample with the addition of calcium hydroxide (B3), which influenced shoot growth more than threefold and root growth almost twofold compared to PM soil. In other cases, calcium addition stimulated test plant growth at a level comparable to the other biocide compounds B1 and B2.

The results of investigating the percentage root growth rate (GFR) and the shoot growth rate (GFS) of the test plants are presented in Table 5.

The analysis of the data presented showed a positive rate of root and shoot growth of plants for test soils S1 and S2. The highest root growth stimulation index GFR > 70% was recorded for soil S1 for mixtures PM(B2) and PM(B3) against *L. sativum.* High GFR values > 60% were obtained for samples PM(B2) and PM(B3) against *L. sativum* for soil S2 and PM(B1) for soil S1. The GFR against *S. alba* for the obtained soil mixtures was considered high and moderate. A very high value of the shoot growth rate GFS 70.1–81.6% was obtained for *L. sativum* excluding PM and PM(B1) mixtures on the S2 soil, at 50.0 and 61.2%, respectively. In the case of *S. alba*, the highest GFS rates were for the mixtures on soil S2 i.e., PM(B2) and PM(B3) of 69.1 and 81.2%, respectively. In all test groups, the results for *S. bicolor* differed significantly from the others and were significantly lower than them. The value of GFR against *S. bicolor* was 10.0–38.6% and that of GFS was 11.6–45.9%. Moreover, it was noted that the plant showed better growth on the S2 soil. The obtained results confirm the positive effect of CaO_2_ action on plant growth stimulation. Research conducted by Małachowska-Jutsz et al. [58] showed that the addition of CaO_2_ at 240 mg/kg to fluoranthene-contaminated soil not only stimulated the effectiveness of the removal of this hydrocarbon, but also improved the root growth of test plants (*L. sativum, S. alba *and* S. bicolor*). It is clear that the reason for better plant growth in biocidal agent-treated soils is the increased amount of oxygen supplied during seed germination, which was also confirmed by other researchers [22,23,24].

## 4. Conclusions

The solid inorganic peroxy compounds (calcium peroxide and sodium percarbonate) are well-known and widely used stimulators of remediation processes in soils contaminated with heavy metals and hardly biodegradable organic compounds. The remediation process is based on the strong action of hydroxyl radicals, one of the most reactive oxidizing agents in nature, which are formed during contact between the inorganic solid peroxy compounds and water. Due to their beneficial effects, they can be used as organic waste additives, providing an alternative to commonly used calcium compounds such as CaO, Ca(OH)_2_ or CaCO_3_. Literature data indicate that the addition of calcium peroxide and sodium percarbonate to fresh poultry manure can effectively reduce harmful pathogens to levels below 1000 cfu/g. The research presented in this article indicates that this is not the only course of action. The use of both compounds as biocidal agents to reduce pathogens in organic fertilizers is associated with the simultaneous stimulation of root and shoot growth of test plants, such as white mustard (*Sinapis alba L.*), peppercorn (*Lepidium sativum L*.) and sorghum (*Sorghum bicolor L. Moench*).

The analysis of water leachates of soils treated with inorganic peroxy compounds showed that the agents increase the bioavailability of components necessary for proper seed germination and plant growth (N, P, K, Ca, Mg and S). In most of the studied cases, the obtained plant shoot and root growth rates were higher for soil mixtures containing organic waste deactivated by biocidal compounds, compared to soils that contained only poultry manure. Nevertheless, it was noted that the presence of sodium percarbonate further stimulated the solubility of heavy metals (As, Pb, Cu, Zn and Cd) present in both soil and organic manures. For this reason, in soils heavily contaminated with heavy metals, instead of 2Na2CO_3_·3H_2_O_2_, it would be advisable to use CaO_2_, which, due to alkalization and the increased presence of calcium in the molecule released as calcium cation, immobilizes the heavy metals, leaving them in the soil as compounds insoluble and inaccessible to plants. The results obtained indicate that phytotoxicity testing should be performed, especially on heavily contaminated soils treated with sodium percarbonate, at a specific time interval after deactivation. Taking into account the existing conditions, calcium peroxide and sodium percarbonate may offer viable options for common calcium compounds such as CaO, Ca(OH)_2_ and CaCO_3_, with these being safe for the environment and very useful for the reclamation of post-industry contaminated soils.

## Figures and Tables

**Table 1:** Physicochemical properties of the soils and poultry manure used in the investigations [37].

Parameter																	
Source	g/kg dm								mg/kg dm								
	TOC	Ca	K	Mg	N	P	Si	Fe	Cd	Co	Cr	Cu	Mn	Ni	Pb	Sr	Zn
PM	418.5	24.0	23.6	8.5	56.7	20.1	1.8	0.8	nd	nd	nd	68.0	383.0	20.0	nd	34.0	428.0
S1	nd	1.6	7.2	0.7	nd	0.3	413.8	11.0	35.2	3.0	12.1	42.3	434.5	10.1	942.5	3.0	6920.1
S2	nd	0.6	4.8	0.2	nd	0.2	441.8	3.7	12.1	5.0	13.1	17.1	133.8	7.0	387.2	4.0	746.3

nd—not determined.

**Table 2:** Physicochemical analysis of water extracts from the tested soils and their blends.

Parameter	Unit	Soil S1					Soil S2				
		S	PM	PM(B1)	PM(B2)	PM(B3)	S	PM	PM(B1)	PM(B2)	PM(B3)
pH	-	6.69	6.23	7.28	6.69	6.67	6.81	6.66	7.68	7.17	7.09
EC	µS/cm	46.00	109.70	257.00	110.9	106.2	54.00	59.3	108.8	85.1	72.5
As	mg/kg dw	0.11	0.52	0.79	0.23	0.25	0.14	0.62	1.48	0.42	0.44
Cd		0.08	0.57	0.73	0.44	0.44	0.06	0.12	0.15	0.15	0.15
Cr		<0.03	0.05	0.09	0.05	0.04	<0.03	0.03	0.04	0.03	0.03
Cu		0.14	0.70	1.71	0.41	0.51	0.15	0.44	0.93	0.45	0.41
Ni		<0.05	0.07	0.11	0.05	0.04	<0.05	0.04	0.04	0.03	0.03
Se		0.13	<0.05	0.01	<0.05	<0.05	<0.05	<0.05	<0.05	<0.05	<0.05
Pb		0.90	9.70	19.26	7.40	6.54	0.82	4.15	5.37	3.44	3.89
Zn		4.30	18.32	19.18	17.66	16.40	13.00	25.62	29.42	28.20	28.13

**Table 3:** ANOVA result for the length of roots the tested plants.

Tested Plant	Experimental Conditions			Experimental Results		
	Soil	SS Effect	Df Effect	MS Effect	F	*p*-Value
*L. sativum*	S1	11,787.69	5	2357.54	166.901	<0.001
	S2	6965.96	5	1393.19	65.934	<0.001
*S. alba*	S1	5699.60	5	1139.92	179.717	<0.001
	S2	14200.6	5	2840.1	59.025	<0.001
*S. bicolor*	S1	1965.69	5	393.14	24.049	<0.001
	S2	2971.07	5	594.21	41.097	<0.001

SS—sum of squares, Df—number of degrees of freedom, MS—mean square, F—statistics, *p*-value—statistically significant if *p* < 0.05.

**Table 4:** ANOVA result for the length of shoots the tested plants.

Tested Plant	Experimental Conditions			Experimental Results		
	Soil	SS Effect	Df Effect	MS Effect	F	*p*-Value
*L. sativum*	S1	11,872.49	5	2374.50	150.104	<0.001
	S2	11,787.69	5	2357.54	166.901	<0.001
*S. alba*	S1	6243.17	5	1248.63	91.385	<0.001
	S2	8759.56	5	1751.91	135.043	<0.001
*S. bicolor*	S1	54.856	5	10.971	2.395	0.044
	S2	207.022	5	41.404	4.0809	0.003

SS—sum of squares, Df—number of degrees of freedom, MS—mean square, F—statistics, *p*-value—statistically significant if *p* < 0.05.

**Table 5:** The growth factors of root (GFR) and shoot (GFS) of tested plants.

Tested Plant		GFR, %			GFS, %		
		*S. alba*	*L. sativum*	*S. bicolor*	*S. alba*	*L. sativum*	*S. bicolor*
S1	PM	56.51	49.69	26.95	52.35	70.08	18.28
	PM(B1)	48.70	61.79	10.00	54.75	79.63	23.05
	PM(B2)	63.28	75.00	29.83	57.26	81.59	11.57
	PM (B3)	56.83	74.36	31.40	46.71	80.49	14.75
S2	PM	37.22	44.59	14.44	27.78	50.00	38.51
	PM(B1)	46.11	53.54	27.81	33.67	61.86	45.94
	PM(B2)	55.15	64.65	38.56	69.15	75.27	43.36
	PM(B3)	67.01	64.71	28.26	81.18	74.72	42.89
	≤25%	very low					
	>25 ≤ 45%	low					
	>45 ≤ 60%	moderate					
	>60 ≤ 70%	high					
	>70%	very high					

## Data Availability

Not applicable.
